# Breaking barriers: widening participation for cross-cultural faculty development in Japan

**DOI:** 10.5116/ijme.6299.c9f6

**Published:** 2022-06-21

**Authors:** Sayaka Oikawa, Maham Stanyon, Shuntaro Aoki, Yoko Moroi, Kiyotaka Yasui, Megumi Yasuda, Takumi Kawai, Yayoi Shikama, Koji Otani

**Affiliations:** 1 Center for Medical Education and Career Development, Fukushima Medical University, Japan

**Keywords:** Faculty development, cultural accessibility, unprofessional behaviour, international faculty, video-based discussion

## Introduction

Participating in faculty development is mandatory for all health professionals involved in medical education to improve their knowledge, skills and teaching practice.^1^ A vehicle to inform and facilitate organisational change, effective faculty development relies on appropriate representation from all groups to capture the broad range of perspectives from teachers at the organisation grass roots.^2^ With rapid globalisation comes the diversification of social needs and the socio-cultural contexts which intersect with medical education, necessitating faculty development programmes that cultivate attendee cultural competence, unconscious bias awareness and an understanding of how these translate to the academic and clinical learning environments. Widening faculty participation through the internationalisation of faculty development programmes is an established method to realise such aims, with successful outcomes documented across diverse contexts.^3^^-^^5^

Internationalisation is a process that is subject to the changing global environment, the most recent influence being the COVID-19 pandemic. The resulting travel restrictions have exerted pressure away from hosting international faculty visiting from abroad to focusing on international diversity within the local faculty.^6^^,^^7^ While a global trend saw the proportion of faculty members from overseas within medical higher education institutions increasing prior to COVID-19, the majority of Japanese universities retained a low ratio of international to local faculty,^8^ a situation which is likely to have worsened due to the pandemic. In addition to low numbers affecting representation, international faculty in Japan also have difficulty participating in local mainstream activities, existing in a 'bubble of foreignness' due to language barriers and cultural challenges.^9^ This feeling of exclusion has been shared by academics in other countries where the number of international faculty members is relatively small,^10^^,^^11^ and also impacts local faculty who may be unsure how to interact smoothly in a domestic multicultural situation. Therefore, in recognising these challenges to international faculty participation and responding to the environmental pressures exerted by COVID-19 in reducing opportunities for international interaction, we have developed a faculty development session which lowers the threshold for the participation of all faculty through increasing cultural accessibility.

The recognition of unprofessional behaviour is a universally important topic which features prominently in faculty development programmes. Although all faculty members engaging in medical education are encouraged to monitor and address unprofessional behaviours encountered among medical students and staff, the recognition and assessment of such behaviours has proven difficult due to unclear definitions of what constitutes unprofessional behaviour,^12^ and contextual differences in social-cultural and ethical frameworks.^13^^-^^15^ Several studies have reported outcomes from cross-cultural faculty development programmes that focus on medical professionalism and found that sharing cultural awareness supported external perspectives among participants, increased identification of culturally learnt patterns and eased the integration of new viewpoints into mainstream practice models.^16^^,^^17^

However, such studies have been done with diverse groups of participants all working within their native contexts with participation as part of a longitudinal programme.^16^^,^^17^ To our knowledge, no previous study has reported on the practical steps necessary to lower the participation threshold for international faculty members in mitigating the cultural and language barriers faced by those working in a non-native setting as a minority group and in supporting local faculty who may struggle in a domestic multicultural setting. We, therefore, developed a faculty development session on the theme of cultivating cross-cultural awareness of unprofessional behaviour at medical school, adapting the session for cultural accessibility, widening participation to include marginalised faculty groups and encourage mutual participation. In this report, we aim to share

the session content and subsequent reflection by the facilitators on the key ideas for international transferability and adoption in future programmes.

### Overview of the faculty development session

The faculty development session was conducted online via Zoom in August 2021 at a Japanese medical school with 16 international faculty members (2.4% of all faculty members), where all official information is communicated in Japanese. Nine medical education faculty members, including a native English-speaking clinical educator from the U.K., were involved in developing the session. We sent a bilingual email, in Japanese and English, announcing the session to the entire faculty, including the 16 international faculty members of our university. In order to encourage the participation of international faculty members, the lead researcher also sent individual personalised emails to these members.

We implemented a lecture and clinical vignettes in a video-based discussion format, which has been shown to be effective in the discussion of context-specific professionalism dilemmas.^3^ In the lecture, we introduced an evidence-based approach to the key concepts of medical students unprofessional behaviour, outlining key differences between Eastern and Western perceptions and conceptual constructions. In this lecture, cultural and contextual factors relevant to the judgement of unprofessional behaviour in a Japanese medical context were introduced. Lecture handouts were written in English, and a Japanese translation was sent in advance to participants. Following the lecture, three video clips created by the facilitators were played as an introduction to the group work. Each video featured an unprofessional behaviour that may be subject to cultural differences in perception and situational management, comprising: 1) a group of students informing their teacher that they would prefer to finish their clinical teaching early due to extra-curricular activities; 2) an attending physician breaking hospital policy in accepting gifts from a patient and sharing them with the students, and 3) an attending physician who undermines a colleague in the presence of medical students and teaches by humiliation. Faculty members used the videos to facilitate small group discussion work via the Zoom breakout room function. In groups of five or six, participants shared their opinions on the behaviours observed, discussing how unprofessional they felt the behaviour to be, contextual aspects, and how international perspectives supported or differed from the Japanese approach to the situation, citing elements of culture in their reasoning guided by a facilitator. Following the group discussion, participants shared their group opinions with the rest of the participants in either Japanese or English. At the end of the session, we collected feedback from the participants via Google forms.

### Feedback from the participants

Forty-one local faculty (6.2% of the total local faculty) and four international faculty members (25% of the total international faculty) participated. Among local Japanese participants, the departments of clinical medicine (18), basic medicine (12), social medicine (10) and the department of laboratory medicine (1) were represented. The international faculty was comprised of members from the departments of clinical medicine (1), basic medicine (2), and social medicine (1), with countries of origin spanning the U.S., Russia, and China. Twenty-nine of 41 participants responded to the post-programme feedback (response rate 70.7%), and 28 respondents (96.6%) rated the faculty development session as "very good" or "good".

### Lessons learnt

We found our online cross-cultural faculty development session on the topic of unprofessional behaviour to be a beneficial and rewarding experience for both international and local faculty in terms of overcoming cultural and linguistic barriers to increase accessibility.

### Breaking language barriers

The bilingual handouts and group work facilitation were practical for smooth communication and ensured mutual participation by the international and local faculty members. In conducting the session within a limited time frame for participants with diverse language proficiencies, we found it effective to prepare materials in both languages and encourage participants to refer back to specific lecture slides in their stronger language, allowing focus on the discussion to be maintained. Discussion in participants' stronger languages enabled both local and international groups to share their opinions freely, deepening the cultural insights revealed about the topic.

### Breaking cultural barriers

In our session, the facilitators included members with cross-cultural experience as educators and clinicians. They played the role of cultural mediators, an essential component for successful international facilitation.^18^ The presence of facilitators with robust knowledge about the topic and practical experience from different cultural contexts allowed an authentic discussion which could be adapted to address participant needs and maximise engagement through facilitator awareness of the participant context and background, which added value to the session.

Video clips effectively minimised language barriers, despite being recorded in Japanese. Supporting this, the use of video illustrating real-life scenarios has been shown to be effective when engaging a culturally and linguistically diverse audience,^19^ and video-based discussion is recognised as an important tool in online medical faculty development programmes.^20^ To achieve the most effective use of video clips, interactive elements promoting learner participation are recommended;^21^ however, incorporating such elements into a multicultural session remained a challenge. In our session, the content of the video clips was based on the real-life experiences of the Japanese facilitators. Thus, the nuances conveyed may have been more effectively picked up by local faculty members during the group work. However, since the videos captured the atmosphere of the situation, including aspects such as actor facial expressions and tone of voice, all participants felt immersed in the situation as if experiencing the event in real-time, allowing international faculty members to relate to the cases with concrete images despite coming from different cultural backgrounds. In addition, we noticed that the contextual aspects triggered discussion points not only among international participants but also among local participants. Because the scenarios were tailored to the Japanese clinical environment, the participants from the departments of basic medicine and social medicine were able to contribute additional perspectives on the unprofessional behaviours observed, improving cultural awareness across speciality boundaries.

### Challenges and difficulties

As session facilitators, we encountered several difficulties. Firstly, we sadly could not accommodate real-time discussion across languages because provision for simultaneous translation was not available. The participants, therefore, reported a brief summary of their group discussion rather than an interactive exchange of ideas in open discussion. Secondly, although we designed the 75-minute evening programme to accommodate those with a heavy clinical schedule and reduce time barriers to attendance, it was not sufficient for participants to gain a deep understanding of each other's mutually complex contexts.

### Conclusions and recommendations

We conducted an online cross-cultural faculty development session for the recognition of unprofessional behaviours at a medical school in Japan. In order to improve cultural accessibility, we introduced several strategies to reduce barriers to participation, including bilingual materials, video clips of real-life scenarios and group discussions facilitated by cultural mediators to give everyone a voice. Through the introduction of basic purposeful steps to improve accessibility, we brought faculty members of different nationalities and languages, as well as different specialities, closer together in authentic discussion united in the goal of recognising cultural differences associated with unprofessional behaviours.

While the internationalisation of medical education principles is a hot topic, clear guidance on approaching the challenges associated with low cultural diversity in faculty development training is lacking. Although we successfully conducted our cross-cultural session through focusing on language and cultural barriers, continuous dialogue with both international and local faculty on obstacles faced when navigating multicultural training could reveal further challenges.

Reflection by facilitators identified further aspects for improvement, such as giving international participants the option of which language to participate in. Although most international faculty is stronger in English, others may prefer Japanese, and likewise, native Japanese speakers may wish to participate in English, particularly while current opportunities to use English in academic settings are limited. Such consideration is important to promote cultural mediation expertise within participant groups and develop an inclusive ethos for future faculty development sessions. The online format may also permit the use of translation tools, which could support participants in future bilingual sessions, mainly if they are not native Japanese or English speakers.

In this report, we show how low numbers of international faculty should not be an excuse for failure to embrace widening access and cultural accessibility within the medical faculty. Diversification is vitally important in recognising and addressing global issues such as unprofessional behaviour where cultural context is highly significant; therefore, increasing participant diversity through encouraging the attendance of marginalised internal faculty groups is an essential goal for future faculty development programmes.

Such changes foster cultural awareness through facilitating new perspectives on culturally sensitive, context-dependent topics within medical education. Widening access to faculty development training in this way not only promotes an atmosphere of inclusivity in training and increased self-awareness but leads the way for concrete and context-sensitive globalisation of the medical education workforce.

### Conflict of Interest

The authors declare that they have no conflict of interest.
